# MCC950 targets the ROS-NEK7-NLRP3 axis to improve type 2 diabetic retinopathy

**DOI:** 10.1038/s41598-025-18438-4

**Published:** 2025-09-23

**Authors:** Kexuan Ren, Xiaofeng Li

**Affiliations:** Dalian University Affiliated Xinhua Hospital, Dalian, 116035 Liaoning China

**Keywords:** MCC950, Concentration gradient, Diabetic retinopathy, ROS-NEK7-NLRP3 axis, Type 2 diabetes mellitus, Vitreous injection, Endocrine system and metabolic diseases, Eye diseases

## Abstract

1 mM of MCC950 targets the ROS-NEK7-NLRP3 axis to ameliorate T2DM in rats and exhibits peak efficacy in improving retinopathy. It has been found that the specific inhibitor MCC950 can alleviate diabetic retinopathy by inhibiting NLRP3 inflammasome, but its concentration-dependent efficacy on retinal pathology needs to be explored. The aim of this study was to quantify the effects of intravitreal injection of graded concentrations of MCC950 (0.01, 0.1, 1,10 mM) on retinal structure and NLRP3 inflammasome signalling in type 2 diabetic male rats, and to reveal that 1 mM MCC950 may exert optimal retinoprotective effects by down-regulating the NEK7-NLRP3 pathway. Type 2 diabetic male rats induced by streptozotocin were administered intravitreal injections of MCC950 at varying concentrations (0.01, 0.1, 1, 10 mM). Quantitative assessments revealed that a concentration of 1 mM MCC950 markedly improved retinal histopathological alterations (*p* < 0.05) and modulated retinal apoptosis and oxidative stress to a considerable degree (*p* < 0.05). On a mechanistic level, MCC950 suppressed NLRP3 inflammasome activation by disrupting the interaction between NEK7 and NLRP3 (manifested by the down-regulation of pathway-associated protein expression, *p*<0.05) and a strong positive correlation between NEK7 and NLRP3 protein expression (*r* = 0.62, *p* = 0.19); inhibited the activation of the NLRP3 inflammasome (manifested by reduced levels of Cleaved Caspase-1, IL-1β, and IL-18, *p* < 0.001). There was a positive correlation between the intensity of ROS fluorescence and the fluorescence expression of NEK7 (*r* = 0.8857, *p* < 0.05), with MCC950 treatment significantly lowering retinal ROS levels at the 1 mM concentration. In conclusion, MCC950 inhibits ROS-mediated NEK7 upregulation, NLRP3 activation, and attenuates pathological damage, oxidative stress, retinal inflammation, and apoptosis in type 2 diabetic retina via ROS-NEK7-NLRP3 pathway.

**Supplementary Information:**

The online version contains supplementary material available at 10.1038/s41598-025-18438-4.

## Introduction

Globally, type 2 diabetes mellitus (T2DM) constitutes more than 90% of all diabetes cases, with diabetic retinopathy (DR) emerging as the predominant microvascular complication, resulting in vision loss in approximately 34.6% of diabetic patients globally, potentially resulting in irreversible visual loss or blindness in its advanced phases^1,2^. NOD-like receptor thermal protein domain associated protein 3 (NLRP3) is one of the key factors triggering retinal vascular inflammation complicated by diabetes mellitus, and NLRP3 initiates cellular focal prolapse by activating cysteinyl aspartate specific proteinase-1 (Caspase-1) via apoptosis-associated speck-like protein containing a CARD (ASC), which promotes the release of the inflammatory factors interleukin 1β (IL-1β) and interleukin 18 (IL-18) and initiates an inflammatory cascade response^3,4^. In contrast to type 1 diabetes(T1DM), DR associated with T2DM is marked by insulin resistance and persistent low-grade inflammation, which may intensify the abnormal activation of the NLRP3 inflammasome^5,6^. Stimulated by danger signals such as high glucose and oxidative stress, NLRP3 promotes the maturation and release of IL-1β and IL-18 by recruiting ASC and caspase-1 to form a complex that drives apoptosis of retinal vascular endothelial cells, disruption of the blood-retinal barrier, pathologic neovascularization, and neuroglial activation, which ultimately leads to irreversible vision loss^7^. Notably, NIMA-associated kinase 7 (NEK7) plays an important role in NLRP3 inflammasome activation^8^: its role as a Ser/Thr kinase during mitotic cell division is a switch between inflammasome activation and cytokinesis, and the interactions between NLRP3 and NEK7 are dependent on either the leucine-rich repeat sequence (LRR) or the NOD of the NLRP3 structural domain and the kinase structural domain of NEK7^9^. Thus, NEK7-NLRP3 binding is required for the assembly and activation of NLRP3 inflammasome. In DR, the mechanisms and mechanisms of NEK7 and ROS interactions remain unexplored, which provides an important avenue for investigating new therapeutic targets^10–13^.

The paucity and ineffectiveness of DR treatments remains a great challenge, with conventional treatments targeting advanced DR such as hypoglycemic agents, retinal laser photocoagulation and anti-VEGF being ineffective or accompanied by significant side effects^14,15^, and vitrectomy being indicated only for end-stage complications such as severe vitreous hemorrhage or traction retinal detachment. The inability of these means to reverse early microvascular inflammatory damage and the lack of direct intervention in NLRP3-driven intrinsic immune activation highlight the urgency of targeting upstream inflammatory pathways.

As a highly specific inhibitor of NLRP3, MCC950 not only inhibits the formation of the ASC complex^16^, but also inhibits IL-1β/IL-18 maturation by binding with high affinity to the ATPase active center of the NACHT structural domain of NLRP3, blocking inflammasome oligomerization and caspase-1 activation^17,18^. In DR, vitreous cavity injection of MCC950 can penetrate the inner border membrane and act directly on the retina, significantly decreasing Müller cell inflammatory factor secretion and repairing the blood-retinal barrier^19,20^. The dosage was selected with reference to the relevant studies from some published articles on experiments of MCC950 in retinal disease models^13,18,19,21–23^, and the equivalent dose was converted in conjunction with the product specification and optimized^24^.

MCC950 has been shown to reduce plaque load by 83% in a mouse atherosclerosis model^25^ but there are still key scientific issues that need to be resolved for its use in DR treatment: (i) most of the existing studies have used a fixed dose (usually 10 mg/kg)^26^, and there is a lack of effective concentration studies targeting localized administration to the retina; and (ii) the mechanism by which MCC950 regulates the NEK7-NLRP3 pathway and its interaction with the ROS pathway is not yet clear.

Compared with the anti-inflammatory effect of MCC950 found by Wang’s group^13^, the present study found that it may attenuate blood-retinal barrier damage by inhibiting the NEK7-NLRP3 axis; this finding is consistent with the findings of Shi’s group^27^ that MC950 may act by disrupting NEK7-NLRP3 interactions, while Zhang’s group^12^ confirmed and found that the NEK7-NLRP3 pathway is involved in the anti-inflammatory effects of MC950. Zou’s group^20^ found that MCC950 reduced ROS production and inhibited retinal apoptosis, and Ge’s group^10^ on DR grouped by disease duration found that MCC950 significantly inhibited retinal IL-1β release and attenuated vascular leakage, but did not specify the exact concentration of the administered drug. In this experiment, a high-fat diet combined with intraperitoneal injection of streptozotocin (STZ) was used to induce T2DM in male SD rats^28–30^(high-fat diet leads to increased adiposity and insulin resistance, and the combination of STZ destroys the pancreatic β-cells and reduces insulin secretion, which together exacerbate the accumulation of lipid and metabolic disorders). For the first time, the concentration of MCC950 was optimized for vitreous injection, and the effect of MCC950 treatment on vascular injury in the retina of T2DM rats was systematically evaluated by up-regulating the NEK7 molecular axis (ROS-NEK7-NLRP3), revealing that the ROS activates the NLRP3 inflammasome, providing a theoretical basis for precise anti-inflammatory treatment.

## Materials and methods

### Reagents and materials

**Table 1:** Contains information regarding the catalogue number of all reagents, and kits used.

Categories	Vendor	Catalog number	Dilution ratio
Primary antibodies NLRP3	Affinity	BF8029	1:1000 (WB)
NEK7	Affinity	DF4467	1:1000 (WB) 1:100 (IF)
ASC	Affinity	DF6304	1:1000 (WB)
Caspase-1	Affinity	AF5418	1:1000 (WB)
Cleaved-caspase-1	Affinity	AF4005	1:1000 (WB)
IL-1beta	Affinity	AF4006	1:1000 (WB)
IL-18	Abmart	M027287	1:1000 (WB)
Bax	Affinity	A19684	1:1000 (WB)
Bcl-2	Affinity	A20777	1:1000 (WB)
Tubulin	Affinity	AF7011	1:1000 (WB)
Secondary antibodies goat anti-mouse IgG	Abcolonal	AS055	1:5000 (WB)
goat anti-rabbit IgG	Abcolonal	AS056	1:5000 (WB)

Reagents	Vendor	Catalog number	
Proteinase K	Beyotime	ST535	
Colorimetric TUNEL Apoptosis Assay Kit	Beyotime	C1091	
BCA protein assay kit	Beyotime	P0010	
ROS assay kit	Sigma	D7008	
Reactive oxygen species assay kit	Beyotime	S0033S	
RIPA Lysis Buffer	Beyotime	P0013B	
Streptozotocin	Solarbio	S8050	
Hematoxylin-Eosin kit	Solarbio	G1120	
MCC950	MCE	HY-12,815 A	
Phenylmethanesulfonyl fluoride	Beyotime	ST505	
PVDF membrane	Millipore	ISEQ00010	
FAS eye fixative solution	Servicebio	G1109	
Phosphatase inhibitor cocktail	Beyotime	P1082	
Antifade mounting medium	Beyotime	P0131	
FGSuper Sensitive ECL Luminescence Reagent	Meilunbio	MA0186	

#### Diabetic rat modeling

Male SD rats, specific-pathogen-free and ranging from 6 to 8 weeks of age with a body weight between 230 and 260 g, were procured from Liaoning Changsheng Biotechnology Co., Ltd., located in Liaoning, China. Each rat involved in this study was guaranteed to be free of pathogens. The number of rats in each experimental group was decided based on our extensive past experience with comparable experimental protocols, as well as data available from other studies. During each experiment, the rats were allotted to various groups at random. All procedures and the care provided to the animals were conducted in strict adherence to the guidelines set forth by the Animal Care and Use Committee, and with the express approval of the Scientific Investigation Board of Xinhua Hospital, which is affiliated with Dalian University in Dalian, China. Every possible measure was taken to alleviate any distress experienced by the animals.

To create a rat model of T2DM^31–34^, the rats underwent a one-week period of acclimatization before being placed on a diet high in sugar and fat (compositionally, 45% of calories from fat) for a duration of 8 weeks. This dietary regimen was applied to all rats except for the normal control group. Subsequently, diabetes was induced through an intraperitoneal injection of streptozotocin (STZ, at a dose of 45 mg/kg) (Table 1)following a 12-hour fasting period. A fasting blood glucose level of 16.7 mmol/L or higher, measured 72 h post-injection, was used as the criterion for the successful induction of T2DM. To expedite the progression of retinopathy, the high-fat diet was maintained for an additional 8 weeks. Meanwhile, the control group was given standard commercial rat chow and water. Blood glucose levels were periodically monitored using tail-vein blood samples and analyzed with yuwell test strips and a glucometer (manufactured by Yuwell Medical Equipment Co., Ltd., Jiangsu, China). Throughout the study, food and water were provided in standard conditions, with body weight and fasting blood glucose levels recorded every two weeks, respectively.

#### Intravitreal injection

Eight weeks after modeling type 2 diabetes, the MCC950 treatment group received intravitreal injections of 2 µL of MCC950 (0.01, 0.1, 1, and 10 mM) in both eyes, with each concentration forming its own group. At the same time, both eyes in the T2DM group received 2 µL vehicle injections as a control. These injections were performed weekly, and after four injections, all subjects were euthanized and their eyes and retinas were subsequently denuded and analyzed. The euthanasia method used in this study was intraperitoneal injection of pentobarbital sodium (50 mg/kg) followed by cervical dislocation, in compliance with institutional animal care guidelines.

#### Hematoxylin-eosin (HE) staining

Following the induction of anesthesia in the rats with pentobarbital sodium (administered at a dose of 50 mg/kg body weight), immediately after removal of the rat eyeballs, they were immersed in FAS eyeball fixative for at least 24 h. The specimens were embedded in wax, cooled, and sectioned into slices of 4 micrometers thickness using a microtome, and the sections were baked in a constant temperature oven at 37 °C for 30 min. After removal, the sections were immersed in xylene I for 10 min, xylene II for 10 min and then in different concentrations of alcohol for 5 min (75%, 85%, 95% and anhydrous ethanol) for gradient dehydration. These sections were subjected to a pretreatment with HD constant staining solution for one minute, followed by hematoxylin dyeing for ten minutes and a water rinse for one minute. Further processing included brief dips in the constant dye solution for ten seconds with subsequent rinsing for twenty seconds, immersion in the constant dye back blue solution for two minutes followed by a water rinse of twenty seconds. The sections were then treated with 95% ethanol for thirty seconds and eosin Y dye solution for five minutes. Finally, the sections were transferred through anhydrous ethanol (three stages of one minute each), xylene I sulfoxide for ten minutes, and xyleneⅡ for an additional ten minutes to achieve transparency, before being mounted with neutral gum.

The specimens were subsequently secured using a neutral adhesive, and subjected to examination under a microscope. Retinal thickness was measured using ImageJ software, including the inner limiting membrane (ILM), nerve fiber layer (NFL), ganglion cell layer (GCL) (quantification of the GCL includes the ILM and NFL), inner plexiform layer (IPL), inner nuclear layer (INL), outer plexiform layer (OPL) and outer nuclear layer (ONL).

#### Determination of ROS

Following humane euthanasia, the rats’ eyes were promptly enucleated. The excised tissues were meticulously embedded in OCT compound, subsequently frozen and sectioned utilizing a cryostat. To assess the retinal ROS levels and oxidative stress, freshly cut retinal cryosections underwent dihydroethidium staining and adhering to the protocol delineated by the manufacturer. Retinal ROS concentrations (sum of reactive oxygen species such as superoxide anion (O_2_^−^)/hydrogen peroxide (H_2_O_2_)) were quantified by the ROS assay kit according to the manufacturer’s instructions. After staining, the sections were sealed with antifade mounting medium and immediately examined under a fluorescence microscope.

#### TUNEL staining

Cell death was measured using TUNEL, according to the manufacturer’s instructions. Retinal sections from different groups were permeabilized by immersion in permeabilizing solution (dissolved in sodium citrate, freshly prepared) and rinsed twice (rinsing times in 5 min). The retinal sections were then stained according to the instructions of the In Situ Apoptosis Detection Kit. The sections were sealed with an antifade mounting medium and immediately observed under a fluorescence microscope.

#### Immunofluorescence

Paraffin sections of rat retina were dewaxed with xylene, rehydrated in graded alcohol solutions including anhydrous ethanol, 95%, 85% and 75% alcohol, blocked with immunostaining sequestering solution. The antigen was retrieved by incubation at 95 ℃ in antigen retrieval solution (0.01 M citrate, pH = 6)for 30 min, after which the slices incubated with primary antibodies (anti-NEK7) at 4℃ overnight, and subsequently incubated with fluorescent-dye conjugated secondary antibodies for 2 h. The sections were sealed with an antifade mounting medium and immediately observed under a fluorescence microscope.

#### Western blotting

The rats were euthanised at the conclusion of the treatment period, and the eyes were extracted expeditiously. The cornea was then meticulously divided along the corneoscleral rim under the microscope. The lens and vitreous were completely expelled, and ophthalmic forceps were inserted into the subretinal cavity to bluntly separate the sclera between the periphery of the retina and the optic papilla. Total protein was extracted from tissues using RIPA lysis buffer containing protease and phosphatase inhibitors. The retina was de-abraded using a tissue grinding pestle and mortar to obtain supernatant proteins, which were quantified using a BCA protein assay kit. Proteins were separated on a 10–15% SDS-PAGE gel and transferred to polyvinylidene fluoride (PVDF) membranes. After being blocked with 5% skim milk, the membranes were incubated with primary antibodies (anti-NLRP3, anti-NEK7, anti-Caspase1, anti-Cleaved-Caspase1, anti-ASC, anti-IL-18, anti-IL-1beta, anti-BAX, anti-BCL-2, anti-Tubulin) at 4℃ overnight, then incubated with secondary HRP-conjugated antibody for 2 h. Blots were then developed using a chemiluminescent kit according to the manufacturer’s instructions. The results were quantified using Image J software (U.S. National Institutes of Health, Bethesda, MD, USA) with Tubulin as an internal control.

#### Statistical analysis

Statistical analyses were performed using IBM SPSS Statistics 30.0(IBM, Armonk, NY, USA). All results are presented as mean ± SEM. One-way ANOVA was applied to compare the data among multiple groups, TUKEY was used for multiple comparisons among sample data, and Pearson was used for correlation analysis. Effect size interpreted according to Cohen’s criteria (*r* > 0.5: large effect). Statistical significance was defined as *p* < 0.05. Graphs were prepared using GraphPad Prism 10(Graphpad, San Diego, CA, USA).

## Results

### Changes in body weight and blood glucose in rats

**Fig. 1:**
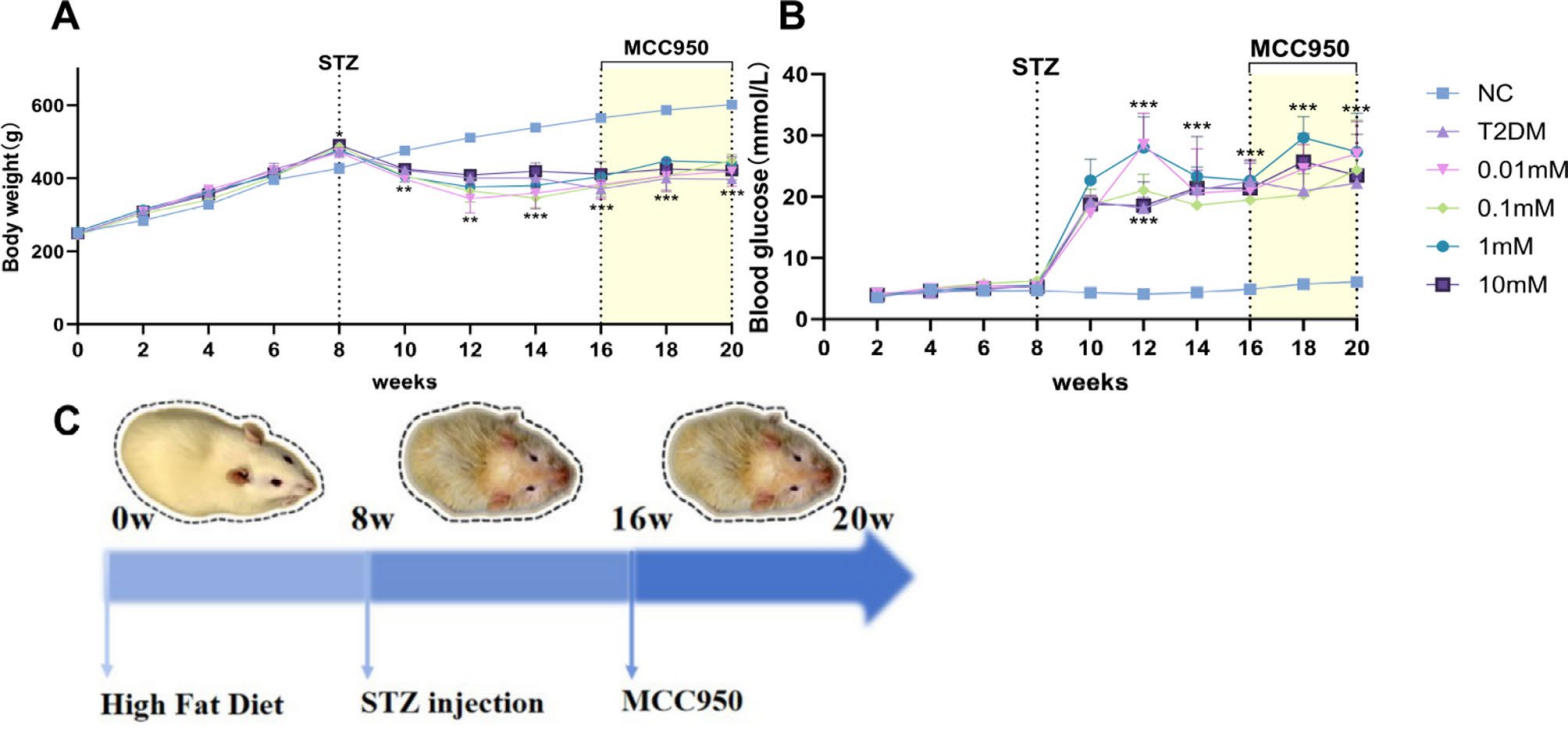
Body weight and blood glucose changes in STZ-induced type 2 diabetic SD rats. **(A)** Body weights of rats in different groups. **(B)** Blood glucose of rats in different groups. **(C)** Model and treatment time axis of type 2 diabetic SD rats. Data are shown as mean ± SEM, *n* ≥ 3 per group. Compared with NC group: *p < 0.05, **p < 0.01,***p < 0.001; NC: normal control group. 0.01,0.1,1,10 mM: levels in T2DM treated with MCC950 at different concentrations (0.01,0.1,1,10 mM).Yellow shading indicates MCC950 treatment period.

Throughout the study period, the body weights and blood glucose levels of the rats were meticulously monitored biweekly. Figure 1A illustrates that the rats in the NC group experienced a steady increase in body weight. Following a 2-week period of acclimatization to the diet, the rats’ blood glucose levels remained within the range of 3.6–4.3 mmol/L. As the rats continued to consume the high-sugar, high-fat diet, both body weight and blood glucose levels of the diabetic rats increased at a faster rate compared to the regular chow-fed NC group, with statistically significant differences starting at week 8. However, after diabetes was induced by intraperitoneal injection of STZ, the rats showed increased blood glucose, urine and water intake, and were significantly thinner than the normal group (Fig. 1A), with dirty, yellowish hair and irritability. These symptoms are consistent with those of diabetes mellitus, indicating that the T2DM model has been successfully constructed. Due to disturbed glucose metabolism, the body is unable to use sugar for energy and instead uses fat. After a large amount of fat is decomposed, this results in emaciation. After the model was established, the blood glucose level of the T2DM group was markedly higher than that of the NC group, and the difference was statistically significant. However, the blood glucose and body weight levels of rats in the MCC950 treatment group did not show statistically significant changes (Fig. 1B), which may be related to the shorter treatment period of MCC950, the lower concentration of MCC950 in the systemic circulation due to the effects of metabolism and excretion of the drug, and the localized effect in the vitreous body rather than the systemic blood circulation).

### Effects of different concentrations of MCC950 on histopathological damage of rat retina

**Fig. 2:**
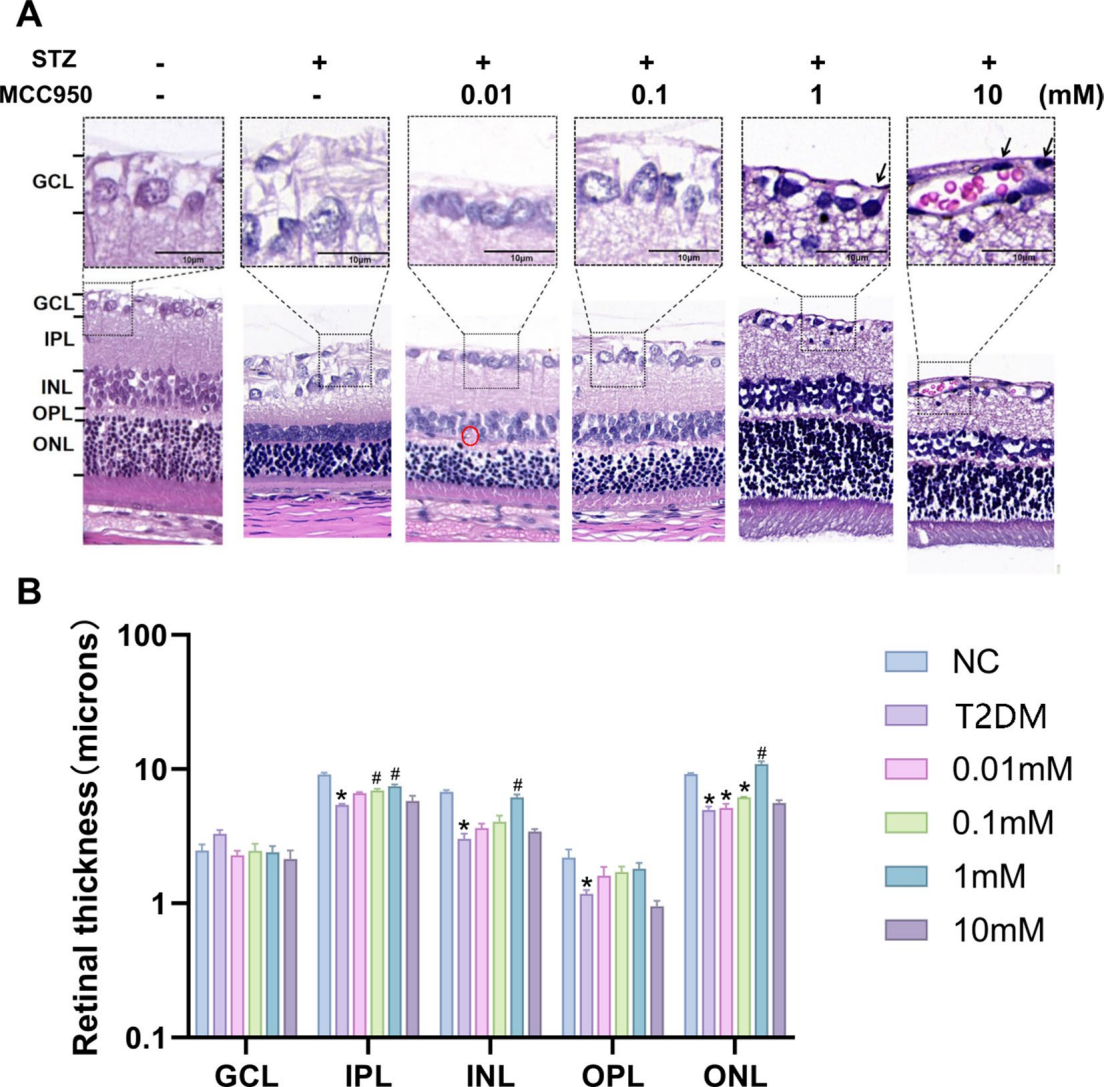
Pathologic structural changes in the retinal tissues of different groups of rats. **(A)** Photographs of HE staining of rats in different groups. **(B)** Analysis of the thickness of the subretinal layers. Data represents mean ± SEM, *n* ≥ 3 per group, Scale bar: 10 μm. Compared with NC group: *p < 0.05; Compared with T2DM group: #*p* < 0.05, ##*p* < 0.01. NC: normal control group.0.01,0.1,1,10 mM: levels in T2DM treated with MCC950 at different concentrations (0.01,0.1,1,10 mM). GCL: Ganglion Cell Layer, IPL: Inner Plexiform Layer, INL: Inner Nuclear Layer, OPL: Outer Plexiform Layer, ONL: Outer Nuclear Layer.

With the progression of T2DM, pathological damage occurred in the rat retina. HE staining showed that the retinal thickness in the NC group was significantly higher than that in the other groups (Fig. 2B). STZ-induced diabetic rat retinas underwent pathological damage: structural disorganization of retinal layers, pre-retinal neovascularization(red circle) and arteriolar thrombosis (Fig. 2A), and a decrease in the retinal ganglion cells. However, the thickness of the GCL did not differ significantly among the groups. There was no significant difference between the groups. Nuclei of pericytes(black arrows) were also seen in the T2DM group. The inner plexiform layer (IPL), inner nuclear layer (INL) and outer nuclear layer (ONL) were thinner than in the NC group, and the MCC950 treatment group improved their thickness. The outer plexiform layer (OPL) was thinner than in the NC group, with no significant difference between the groups. Compared with the T2DM group, the structure of the inner plexiform layer (IPL), inner nuclear layer (INL) and outer nuclear layer (ONL) was significantly improved under the treatment of 1 mM concentration of MCC950, and the cells of the IPL and ONL tended to be neatly aligned, and the thickening of the vascular basement membrane and exudative lesions were significantly reduced.

### Effects of different concentrations of MCC950 on apoptosis in rat retinal tissues

**Fig. 3:**
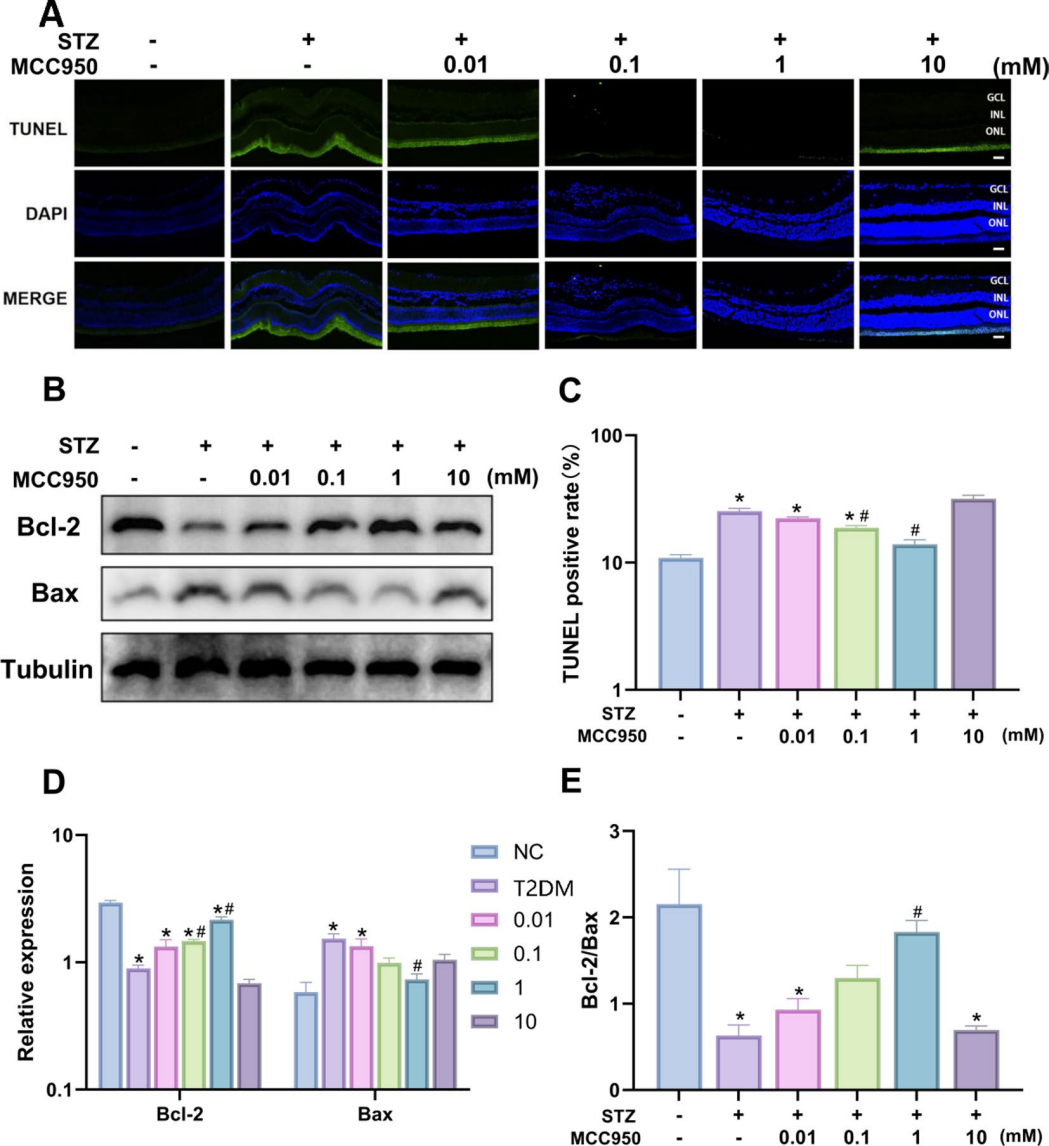
Effect of MCC950 on STZ-induced apoptosis in retinal tissues of type 2 diabetic SD rats. **(A**,** C)** Detection of apoptotic cells in different groups of rat retina by TUNEL method. **(B-E)** Displayed is the expression of Bax and Bcl-2 in rat retina in different groups. Tubulin served as an endogenous reference for normalization.Data represents mean ± SEM, *n* ≥ 3 per group, Scale bar: 50 μm. Compared with NC group: *p < 0.05; Compared with T2DM group: #*p* < 0.05. NC: normal control group.0.01,0.1,1,10 mM: levels in T2DM treated with MCC950 at different concentrations (0.01,0.1,1,10 mM).

In order to evaluate the effects of different concentrations of MCC950 on the apoptosis of retinal tissue cells, we detected the apoptosis of retinal tissue cells in rats by TUNEL method. The results showed that the retinal apoptosis in STZ-induced diabetic rats was markedly increased, Different concentrations of MCC950 (0.01, 0.1, 1, 10 mM) had different degrees of effects on apoptosis induced by prolonged hyperglycemia in retinal tissues, and the concentration response peaked at 1 mM - retinal apoptosis was significantly improved, and the difference was statistically significant(Fig. 3A, C). Western blot showed that Bax expression was significantly elevated and Bcl-2 was significantly decreased in the retina of T2DM rats, and the difference was statistically significant. The increase in the Bax/Bcl-2 ratio drove apoptosis in retinal ganglion cells and vascular endothelium through activation of the caspase-3 cascade reaction, and thus exacerbated the disruption of the blood-retinal barrier. After treatment with MCC950, the expression of the anti-apoptotic protein Bcl-2 was up-regulated and the expression of the pro-apoptotic Bax protein was down-regulated, leading to an increase in the Bcl-2/Bax ratio, which further confirmed its anti-apoptotic effect (Fig. 3D-E).

### Effects of different concentrations of MCC950 on ROS expression in rat retinal tissues and correlation analysis with NEK7 expression

**Fig. 4:**
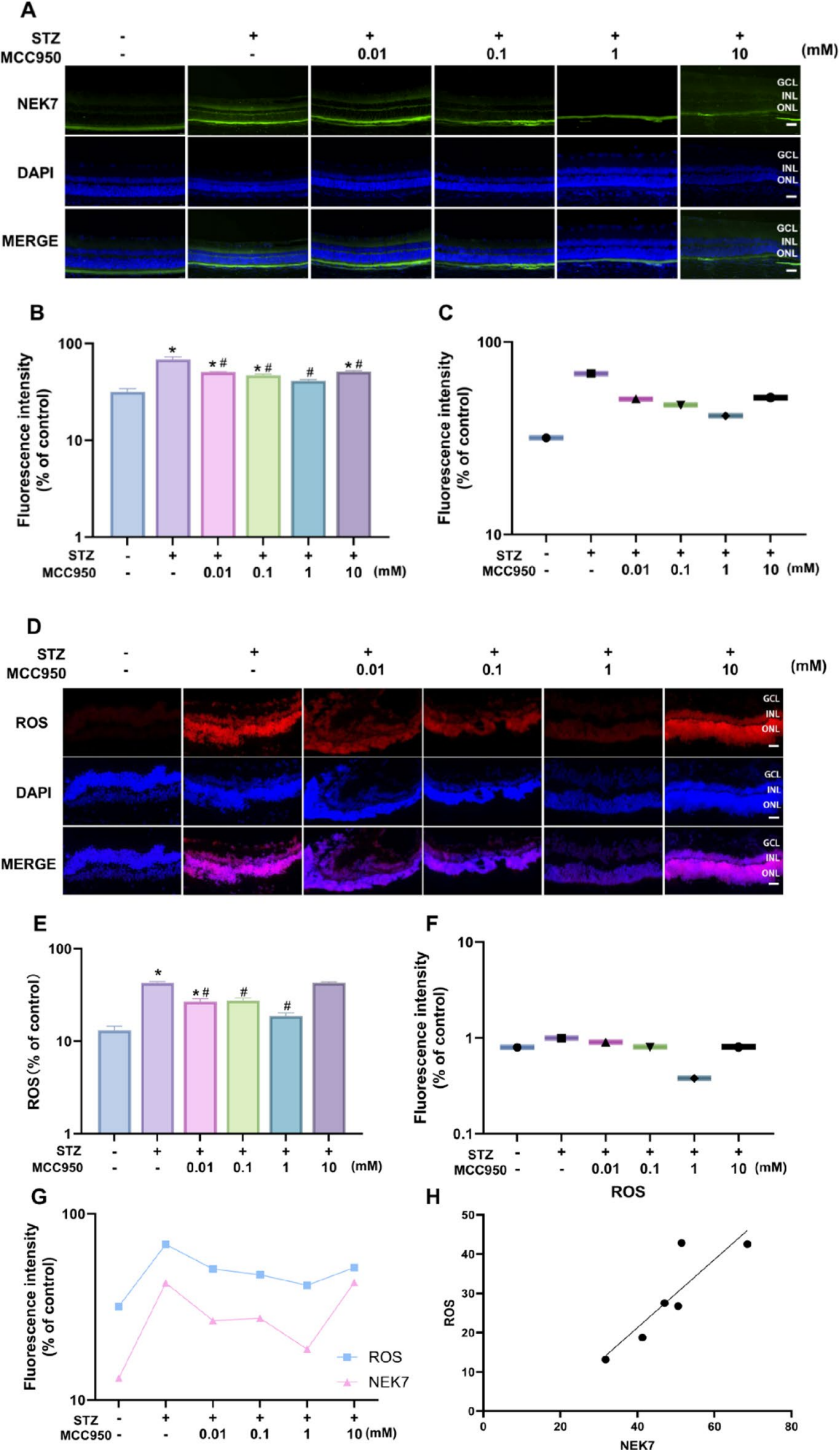
Analysis of the fluorescence intensity. **(A-C)** Immunofluorescence analysis of the expression of NEK7 in rat retina in different groups. **(D-F)** Immunofluorescence analysis of the expression of ROS in rat retina in different groups. **(G-H)** Correlation analysis between ROS and NEK7 in retina of rats. *n* ≥ 3 per group; Data represents mean ± SEM, *n* ≥ 3 per group, Scale bar: 50 μm. Compared with NC group: *p < 0.05;Compared with T2DM group: #*p* < 0.05. NC: normal control group.0.01,0.1,1,10 mM: levels in T2DM treated with MCC950 at different concentrations (0.01,0.1,1,10 mM).

In order to verify whether MCC950 can block the NEK7/NLRP3 pathway in diabetic retinopathy by binding specifically with NLRP3 and thus achieve the therapeutic effect, we used immunofluorescence localization (Fig. 4A) to analyze the NEK7 expression level in the retina. The results showed that in the NC group, the NEK7 immunofluorescence signal (green) was mainly localized in the ganglion cell layer (GCL) and the inner nuclear layer (INL) of the retina, and was weakly expressed, i.e., the basal expression level of NEK7 was low in the physiological state. In the T2DM group, the fluorescence signal of NEK7 was significantly enhanced and widely distributed in the ganglion cell layer, inner nuclear layer and perivascular area. Quantitative analysis showed that the fluorescence intensity was significantly higher in the T2DM group than in the NC group (*p* < 0.001, Fig. 4B), indicating that diabetic pathology induced the up-regulation of NEK7 expression.In the MCC950 concentration gradient treatment groups, the expression levels of NEK7 were reduced to different degrees. Among them, the 1 mM concentration inhibited NEK7 expression most significantly (Fig. 4B, D, E), suggesting that MCC950 may have reversed the abnormal activation of NEK7 induced by T2DM.

In addition, to verify the protective effect of concentration gradient MCC950 against apoptosis and oxidative damage in the retina of diabetic rats in the T2DM group, we detected the expression of ROS in the retinal tissues of rats in each group and further analyzed the correlation between ROS and NEK7 expression. The results suggested that compared with diabetic rats in the T2DM group, ROS accumulation was reduced under treatment in the 10 mM concentration group, but there was no statistically significant difference; in the concentration range of 0.01 mM to 1 mM, the expression of ROS gradually decreased with the increase of the concentration of MCC950, and the expression was the lowest at the 1 mM concentration (*p* < 0.001), and the difference was statistically significant (Fig. 4C, D,F). Pearson correlation analysis showed a significant positive correlation between NEK7 fluorescence intensity and retinal ROS levels in rat retinal tissues (*p* < 0.05,*r* = 0.8857, Fig. 4G), suggesting that NEK7 plays an important role in regulating oxidative stress in the retina and verifying the activation of NLRP3 inflammasome by ROS through upregulation of NEK7.

### Gradient MCC950 inhibits NEK7/NLRP3 pathway-related protein expression

**Fig. 5:**
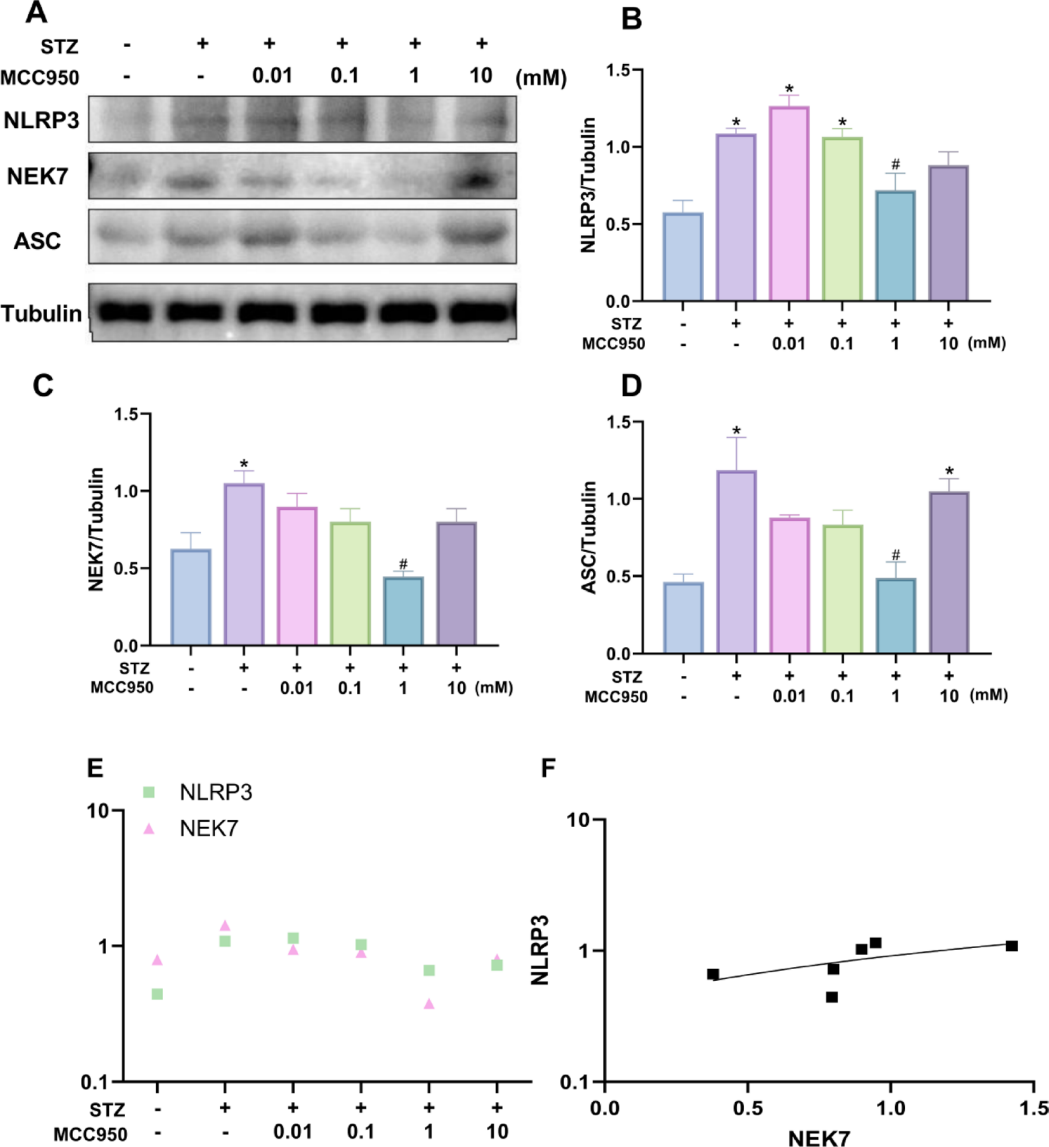

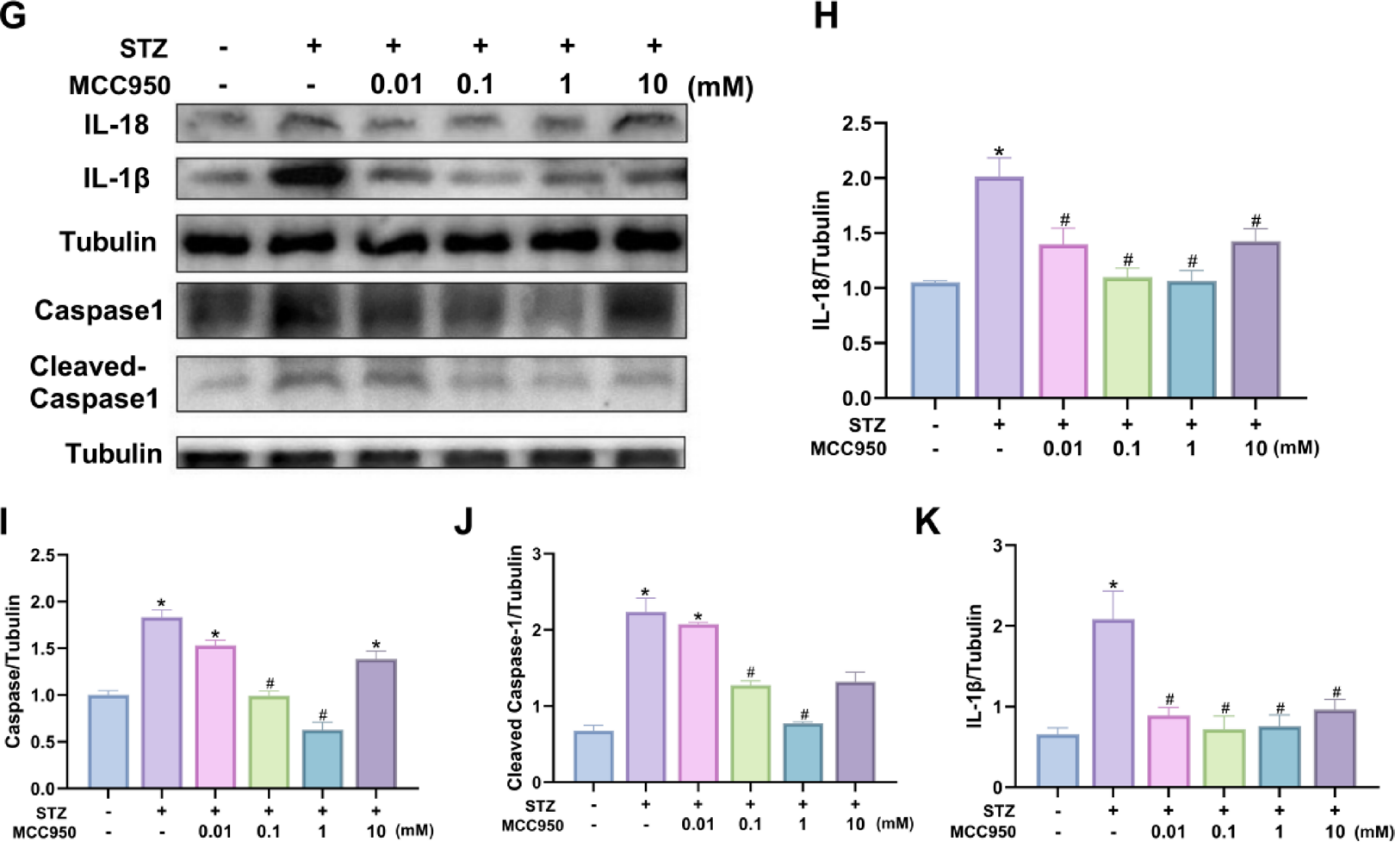
MCC950 inhibited the activation of NLRP3 inflammasome by western blot analysis. **(A-D)** The expression of NLRP3、NEK7 and ASC in rat retina in different groups. **(E-F)** Correlation analysis of NLRP3 and NEK7 expression in retinal of rats. **(G-K)** The expression of inflammatory factor Caspase1 and Cleaved-Caspase1, IL-18 and IL-1beta in rat retina in different groups.Tubulin served as an endogenous reference for normalization.Data represents mean ± SEM, *n* ≥ 3 per group. Compared with NC group: *p < 0.05;Compared with T2DM group: #*p* < 0.05. NC: normal control group.0.01,0.1,1,10mM: levels in T2DM treated with MCC950 at different concentrations (0.01,0.1,1,10mM).

To clarify the effects of different concentrations of MCC950 on the NEK7/NLRP3 pathway in the rat retina, we examined the expression of NLRP3, NEK7 and ASC proteins by Western blot. ASC (puncta-like protein) plays a central bridging role in the activation of NLRP3 inflammasome: its PYD structural domain binds to NLRP3, and the CARD structural domain raises caspase-1, which drives inflammasome assembly. The experimental results showed that the expression of NLRP3, NEK7 and ASC proteins was significantly elevated in the retinal tissues of rats in the T2DM group compared with the NC group (*P* < 0.05). MCC950 intervention inhibited the expression of the abovementioned proteins, with the most significant inhibition effect in the 1 mM concentration group (*P* < 0.05, Fig. 5A-D).

Quantitative analysis revealed a strong positive correlation between NEK7 and NLRP3 protein expression in diabetic retinas (Pearson’s *r* = 0.62, R² = 0.38). Despite limited sample size constraining statistical significance (*p* = 0.19), the large effect size (Cohen’s q = 0.72) aligns with their established functional interaction. The synergistic role of the NEK7-NLRP3 complex in the pathologic process of DR was further confirmed (Fig. 5E-F).

### Effect of different concentrations of MCC950 on the expression of inflammatory factors in rat retina

To verify the effect of different concentrations on each inflammatory factor in rat retina, the expression of inflammatory factors Caspase-1, Cleaved-Caspase-1, IL-1β and IL-18 in all rat retinal tissues were detected by Western blot. The results suggested that the expression of inflammatory factors was markedly increased in diabetic rats in the T2DM group compared to NC, and the differences were all statistically significant. When different concentrations of MCC950 were administered to the vitreous cavity of T2DM rats, the release of the inflammatory factors, Caspase-1, Cleaved-Caspase-1, IL-1β, and IL-18, was reduced to varying degrees and reached a minimum at a concentration of 1 mM(Fig. 5G-K).

## Discussion

DR is characterized by chronic inflammation-driven progressive retinal vascular injury, leading to blood-retinal barrier disruption, pathologic neovascularization, and neurodegeneration, ultimately leading to irreversible vision loss. The high glucose-induced ROS-NEK7-NLRP3 signaling axis is the central mechanism driving early vascular injury in DR: oxidative stress drives the formation of the NEK7-NLRP3 complex, which in turn assembles and activates inflammasome to damage the retina. Hyperglycemia triggers a retinal ROS burst through polyol pathway activation and accumulation of AGEs^33^, which in turn phosphorylates the Tyr97 site of NEK7 kinase and promotes the binding of NEK7 to the LRR structural domain of NLRP3^35,36^, resulting in the formation of a stable NEK7-NLRP3 complex^9^. The complex acts as a core sensor of damage-associated molecular patterns (DAMP), recruiting ASC junction proteins and activating caspase-1^12,17,37^, and the NACHT structural domain serves as the active center of ATPase activity of NLRP3, driving oligomerization through hydrolysis of ATP^16^, and the conformational change of its WalkerA/B motifs is the rate-limiting step in the assembly of inflammasome. Activated caspase-1 mediates retinal damage by promoting vascular endothelial pyroptosis and releasing mature IL-1β/IL-18 dual pathways^7,38–40^. In conclusion, the hyperglycemia-induced ROS-NEK7-NLRP3 axis orchestrates the cascade of molecular events that drive retinal damage in DR. It provides new perspectives for understanding the pathogenesis of DR and points the way to developing targeted therapeutic strategies.

Core therapies have their own limitations, such as limited anti-VEGF response rates and laser damage to the visual field. Clinical needs are unmet with neuroprotective and inflammatory targeting. Emerging therapies include NLRP3 inhibitors, gene therapy, neuroprotective agents, and oral multi-targeted agents. However, only MCC950 relieves early inflammation in DR. Several studies have validated the therapeutic potential of MCC950. Under high glucose conditions, it not only inhibits apoptosis of Müller cells and human retinal endothelial cells (HRECs) while maintaining the integrity of the blood-retinal barrier^20,41^, but also blocks NEK7 - NLRP3 interactions^12^ and down-regulates inflammatory factor levels^13,21,27^. Vitreous cavity injection of MCC950 significantly reduces vascular leakage and reverses high glucose-induced retinal histological damage^10,21^. The present study further confirmed the effectiveness of MCC950 in inhibiting retinal apoptosis, inflammatory response, and repairing retinal structure, providing a more adequate experimental basis for its application in DR treatment.

In this study, a T2DM model was successfully constructed, and the rats showed typical diabetic signs, such as elevated blood glucose, polyphagia, polydipsia and weight loss. This was due to the selective destruction of pancreatic β-cells after intraperitoneal injection of STZ, which led to insufficient insulin production and elevated blood glucose levels, thereby disrupting normal metabolic functions. However, MCC950-treated T2DM rats did not show statistically significant changes in blood glucose and body weight levels, suggesting that MCC950 selectively binds to the ATPase structural domain of NLRP3 to block the assembly of inflammasome and does not interfere with key nodes of the insulin signaling pathway at therapeutic doses (0.01-10 mM), and therefore does not exacerbate the core pathology of T2DM when treating DR --insulin resistance, which is consistent with the findings of liu’s group^42^. In addition, this may be related to the shorter treatment period of MCC950, the effect of drug metabolism and excretion, and the low concentration of drug entering the systemic circulation after intravitreal injection.

We used intravitreal injection as the route of administration, which allows us to penetrate the inner rim of the membrane and act directly on the retina, significantly decreasing the secretion of inflammatory factors in the retina and repairing the blood-retinal barrier^19,20^, as well as reducing the systemic side effects^41,43^. Although previous animal studies have used oral MCC950 at 0.3–10 mg/kg^26,44–46^ or intravitreal injections of 1 µM-10 mM^13,18,21,23,24^, these concentrations are specific to particular disease models. Through optimization based on retinal disease studies^19,22^ and dose conversion^24^, we determined that 0.01-10 mM was the optimal screening range. It was found that 1 mM was the optimal vitreous injection concentration. At this dose, MCC950 achieves effective intravitreal therapy while maintaining a small systemic exposure, greatly reducing potential adverse effects. MCC950 provides a more comprehensive protection by simultaneously alleviating early DR inflammation, apoptosis and oxidative damage. Notably, the reduced efficacy observed at a concentration of 10 mM may be attributed to negative feedback activation at too high a concentration, nonspecific effects, or metabolic factors^44^.

In terms of structural and functional changes in the retina, HE staining showed a significant reduction in the thickness and structural disorganization of the retinal layers in the T2DM group, which is an important structural basis for the progression of DR. In contrast, the retinal structure was significantly repaired in the MCC950 treatment group at a concentration of 1 mM (Fig. 2), which was consistent with published studies^10,21^, indicating that MCC950 had a protective effect on retinal tissues. In terms of apoptosis, TUNEL staining and protein blotting assay confirmed a significant increase in the apoptosis rate of retinal tissues and a decrease in the Bcl-2/Bax ratio in the T2DM group, which further exacerbated the damage of retinal tissues. However, treatment with 1 mM of MCC950 reversed this trend (Fig. 3), suggesting its anti-apoptotic effect, which is consistent with the findings of Zou’s group^20^ and Zhang’s group^12^.

This study focused on the role of ROS-NEK7-NLRP3 axis in DR. The results showed that the fluorescence intensity of NEK7 and ROS in the retina was significantly enhanced in the T2DM group, and the two showed a strong correlation (*r* = 0.82, Fig. 4), suggesting that high glucose-induced ROS accumulation in the retina upregulated the expression of NEK7. Meanwhile, Western blotting assay revealed activation of the NEK7-NLRP3 pathway accompanied by elevated levels of IL-1β and IL-18, and there was also a correlation between the expression of NEK7 and NLRP3 (Fig. 5). In contrast, 1 mM of MCC950 was able to attenuate oxidative stress and inhibit the activation of this pathway and down-regulate the expression of inflammatory factors, suggesting an anti-inflammatory effect, which is consistent with the findings of published studies^12,13,27^.

This study demonstrated that specific blockade of the NLRP3 inflammasome by MCC950 effectively inhibited the inflammatory response, ameliorated histopathological changes, and reduced oxidative stress, while reversing retinal apoptosis. This is consistent with previous studies^12,20,47^. MCC950 intervenes in DR pathological processes through a synergistic multi-targeted effect: (i) specifically binds to the WalkerB motifs in the NACHT structural domain, competitively inhibiting NEK7 binding and disrupting the formation of the NEK7-NLRP3 complex, thereby inhibiting the assembly of the NLRP3 inflammasome^16,17,25,34^; (ii) it reduces retinal ROS levels by upregulating SOD1 activity, breaking the vicious cycle between oxidative stress and inflammation. Compared with single-target inhibitors, this multi-target mode of action gives MCC950 a clear advantage in the treatment of early DR.

However, there are limitations in the study that need to be addressed in the future: direct validation of NEK7-NLRP3 binding by co-ip, development of improved delivery systems (e.g., extended-release formulations) to prolong efficacy, and the need to validate the long-term effects of MCC950 on the blood-retinal barrier.

## Conclusions

MCC950 (1 mM intravitreal injection) targeting the NACHT structural domain provides a mechanism-driven therapeutic strategy for DR by blocking the ROS-NEK7-NLRP3 axis.

## Supplementary Information

Below is the link to the electronic supplementary material.

Supplementary Material 1

## Data Availability

Data will be made available on request.The datasets generated and analyzed during the current study are available from the first author upon reasonable request.
