# Supplementary figures and images for: MCC950 targets the ROS-NEK7-NLRP3 axis to improve type 2 diabetic retinopathy
Source: Sci Rep. 2025 Sep 23;15:32637. doi: 10.1038/s41598-025-18438-4 (PMC12457656; doi:10.1038/s41598-025-18438-4)

Supplementary Raw Blot Figure

Fig.3B:

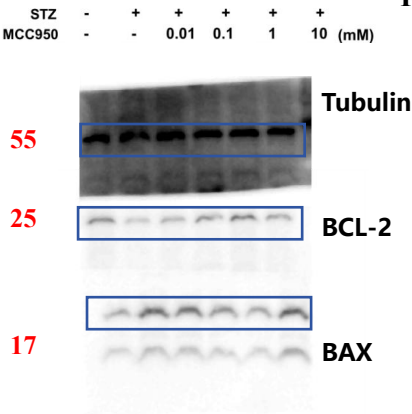

Fig.5B:

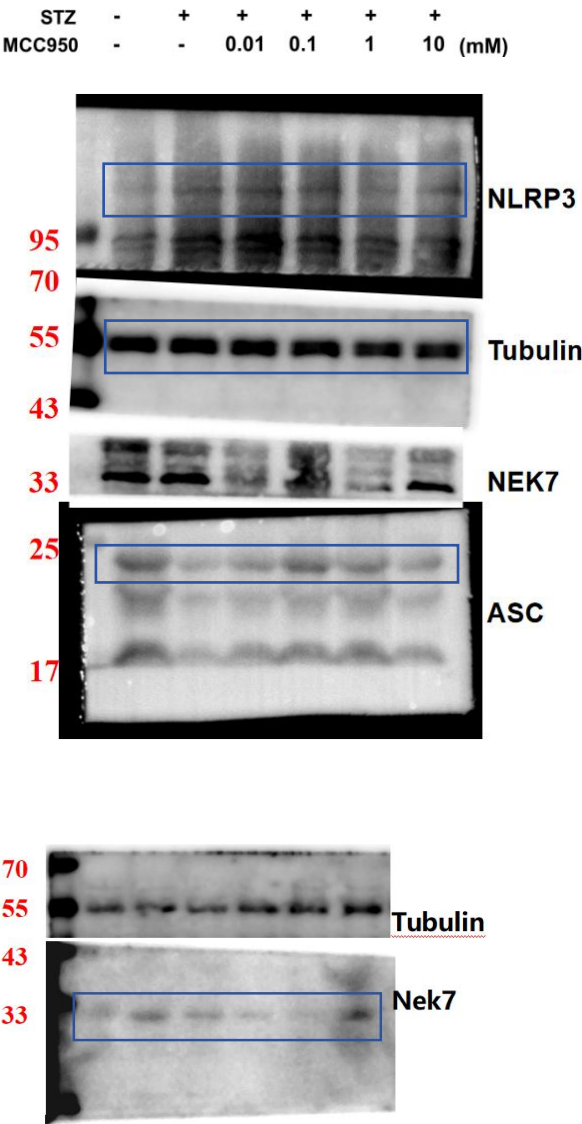

Fig.5G:

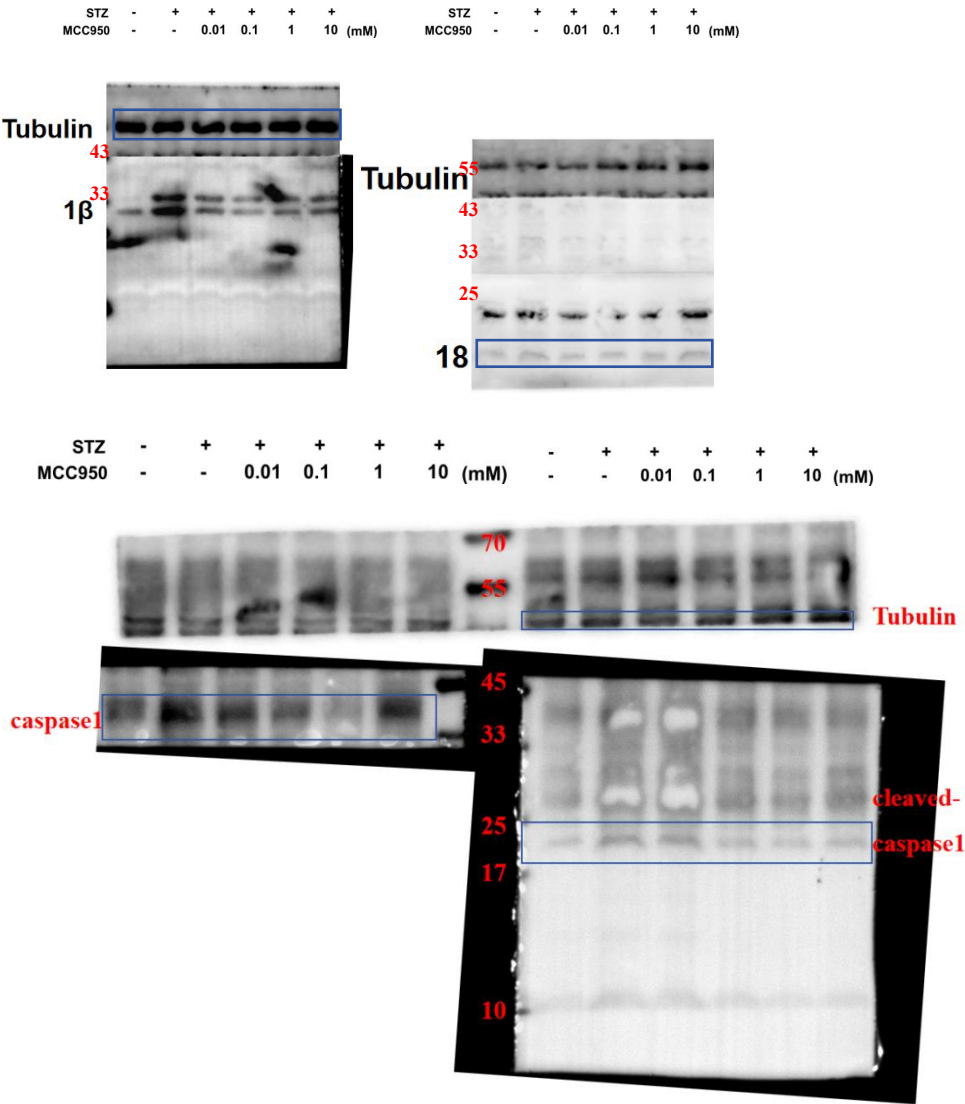

Supplement: Supplementary file 1 — Supplementary Material 1 [file 41598_2025_18438_MOESM1_ESM.pdf]
